# Teaching basic trauma: validating FluoroSim, a digital fluoroscopic simulator for guide-wire insertion in hip surgery

**DOI:** 10.1080/17453674.2018.1466233

**Published:** 2018-05-10

**Authors:** Kapil Sugand, Robert A Wescott, Richard Carrington, Alister Hart, Bernard H Van Duren

**Affiliations:** a Institute of Orthopaedics & Musculoskeletal Sciences, University College London, London, UK;;; b MSk Lab, Imperial College London, Charing Cross Hospital, London, UK;;; c Royal National Orthopaedic Hospital, Stanmore, London, UK

## Abstract

Background and purpose — Simulation is an adjunct to surgical education. However, nothing can accurately simulate fluoroscopic procedures in orthopedic trauma. Current options for training with fluoroscopy are either intraoperative, which risks radiation, or use of expensive and unrealistic virtual reality simulators. We introduce FluoroSim, an inexpensive digital fluoroscopy simulator without the need for radiation.

Patients and methods — This was a multicenter study with 26 surgeons in which everyone completed 1 attempt at inserting a guide-wire into a femoral dry bone using surgical equipment and FluoroSim. 5 objective performance metrics were recorded in real-time to assess construct validity. The surgeons were categorized based on the number of dynamic hip screws (DHS) performed: novices (< 10), intermediates (10–39) and experts (≥ 40). A 7-point Likert scale questionnaire assessed the face and content validity of FluoroSim.

Results — Construct validity was present for 2 clinically validated metrics in DHS surgery. Experts and intermediates statistically significantly outperformed novices for tip–apex distance and for cut-out rate. Novices took the least number of radiographs. Face and content validity were also observed.

Interpretation — FluoroSim discriminated between novice and intermediate or expert surgeons based on tip–apex distance and cut-out rate while demonstrating face and content validity. FluoroSim provides a useful adjunct to orthopedic training. Our findings concur with results from studies using other simulation modalities. FluoroSim can be implemented for education easily and cheaply away from theater in a safe and controlled environment.

Orthopedic training has declined after the introduction of the European Working Time Directive (EWTD) in 2004, leading to fewer operative training hours, a reduction from 30,000 to 15,000 hours (Temple 2010). A reduction in training time, seen on both the European and North American continents, has been perceived negatively in surgical education ­(Zuckerman et al. 2005, Egan et al. 2012). Junior trainees are taking longer to complete operations (Wilson et al. 2010), which reduces theatre efficiency and increases economic burden.

Stable extracapsular neck of femur (NOF) fractures make up a significant proportion of all hip fractures and are treated using a dynamic hip screw (DHS) (Utrilla et al. 2005). Other alternatives include compression or cannulated hip screws. The DHS implant may fail due to cut-out, reported to be between approximately 2% and 7% (Chirodian et al. 2005, Hsueh et al. 2010), predicted by the tip–apex distance (TAD) (Andruszkow et al. 2012).

Fluoroscopy is used during the DHS procedure (Baratz et al. 2014), which carries radiation risks. Inexperienced trainees take more images, thus increasing radiation exposure (Khan et al. 2012). Digital imaging alternatives have been explored clinically, but are not used for training or simulation (Grutzner and Suhm 2004).

Current DHS simulation options consist of virtual reality (VR) or workshop dry bones (Akhtar et al. 2015). VR DHS simulation enables trainees to learn the cognitive process of the DHS procedure with the help of digital fluoroscopy, but at the expense of not using actual equipment to practice manual dexterity. Workshop dry bone simulation develops motor skills as used in theatre; however, fluoroscopy is not used due to the radiation risks (Stirling et al. 2014).

We have developed a digital fluoroscopic simulation system, FluoroSim, that produces realistic radiographs for simulation without using radiation. Both cognitive and motor skills needed for the insertion of a DHS guide-wire can be developed by giving real-time feedback through 5 objective performance metrics.

We aim to demonstrate whether FluoroSim can: 1) separate surgeons with different levels of surgical experience using 5 objective metrics (construct validity); 2) offer realistic steps of a guide-wire insertion into a hip (content validity); and 3) offer realistic radiographs using FluoroSim (face validity). 

## Materials and methods

### Setup

**Figure 1.**
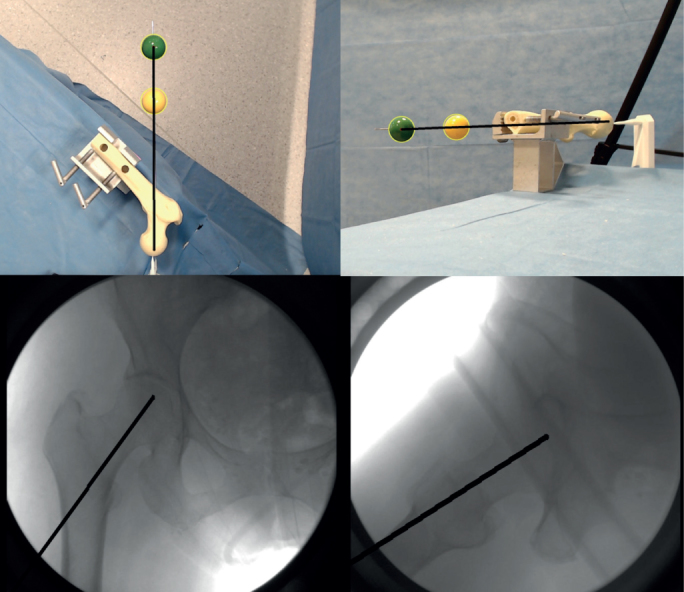
Control screen of the FluoroSim software running with the calibration femur. The software locates the colored markers and finds their center. It marks the position of the guide-wire on the camera image and, using the ATM, overlays this onto the pre-loaded radiograph. Both AP and CTL images are produced.

FluoroSim is an augmented-reality imaging and targeting software that uses 2 Logitech c920 cameras (Logitech, Romanel-sur-Morges, Switzerland) to track 2 colored markers attached to a DHS guide-wire (van Duren et al. 2018). The system is calibrated using a workshop femur (3B Scientific, Hamburg, Germany). With the guide-wire inserted into the femur, 3 points from the digital camera image are selected and matched to 3 corresponding points on a pre-loaded hip radiograph in both the anterior-posterior (AP) and cross-table lateral (CTL) plane (to produce an affine transformation matrix). Image processing algorithms locate the center of the markers on the DHS guide-wire and overlay its position onto the radiograph (Figure 1).

**Figure 2.**
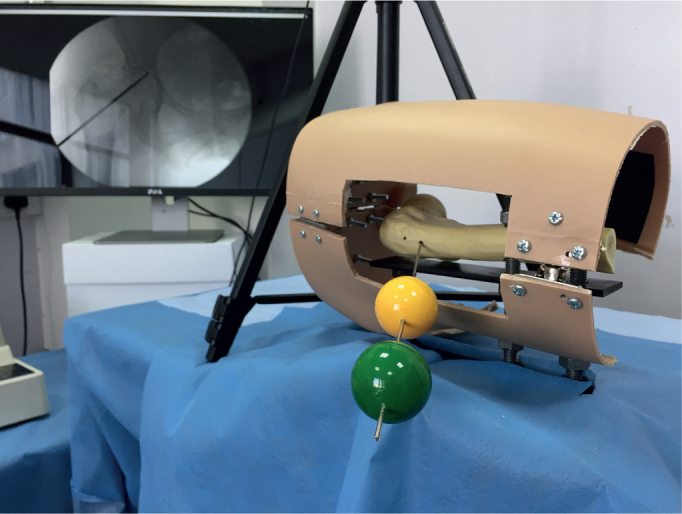
A right phantom limb produced out of a mannequin leg with an interchangeable workshop femur. In the background of the image the simulated radiograph of this construct may be seen.

A simulation scenario was set up using the FluoroSim software run on a MacBook Pro with macOS Sierra 10.12.1 (Apple Inc., Cupertino, CA, USA) for digital imaging. A phantom limb model represented a right hip which was draped, produced from a hollow polyethylene mannequin and interchangeable workshop femurs (Figure 2). A Stryker system 4 rotary drill (Stryker, Kalamazoo, MI, USA) and a 135-degree angle guide with guide-wire were used for high-fidelity immersive simulation. This equipment in total cost less than $3,000, a fraction of the price of commercial virtual reality fluoroscopic simulators.

### Objective metrics

The FluoroSim software calculated real-time objective performance metrics including: 1) TAD (mm); 2) COR (%) according to Baumgaertner’s curve (Baumgaertner et al. 1995); 3) total procedural time (s); 4) total number of radiographs; and 5) total number of guide-wire retries.

### Subjective metrics

All cohorts assessed the face and content validity of FluoroSim. A 7-point Likert scale questionnaire inquired as to agreement with 4 statements regarding the realistic appearance of FluoroSim and its usefulness for training.

### Logistics

**Figure 3.**
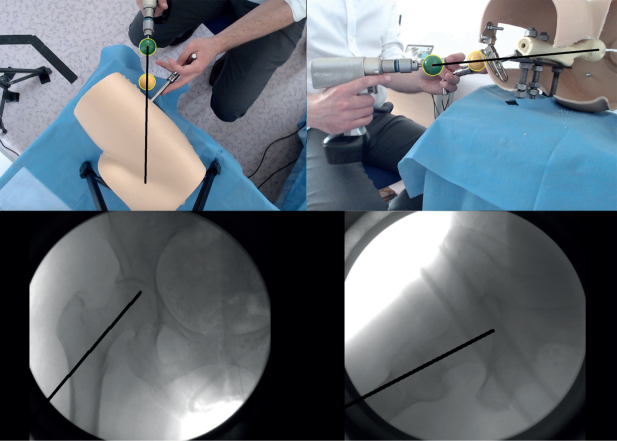
A surgeon using FluoroSim with the phantom limb, surgical equipment and the imaging system.

26 surgeons from Northwick Park (London, UK), Central Middlesex (London, UK), and the Princess Alexandra Hospital (Harlow, UK) were recruited voluntarily and categorized into 3 groups based on the number of DHS procedures performed: novices (< 10), intermediates (10–39) and experts (≥ 40). Each participant received a standardized explanation of the task (Table 1) and then had 1 attempt to insert the DHS guide-wire using FluoroSim for AP or CTL views (Figure 3). The 5 objective metrics were recorded at the end of each attempt.

**Table 1.** Checklist used to standardize the participants’ instructions

Standardized instruction checklist
1. Explain the basic working of FluoroSim, highlighting the importance of not covering the tracking markers or bending the guide-wire.
2. Explain the 5 objective metrics recorded.
3. Explain the main goal of the task: To achieve optimal guide-wire placement as if they were completing a DHS procedure thus giving them an optimal TAD.
4. Highlight that time was being recorded but the focus was on achieving an optimal guide-wire placement.
5. Explain that they should indicate when they are happy with their final guide-wire placement.

### Inclusion/exclusion criteria

Inclusion criteria included having observed at least 1 DHS procedure in theatre. Exclusion criteria consisted of having attempted DHS simulation beforehand, undergraduates, and non-orthopedic trainees.

### Statistics

The data were analyzed in SPSS (version 24.0, IBM Corp, Armonk, NY, USA). Normality was checked using histograms and Shapiro–Wilk testing at α = 5%.

**Figure 4.**
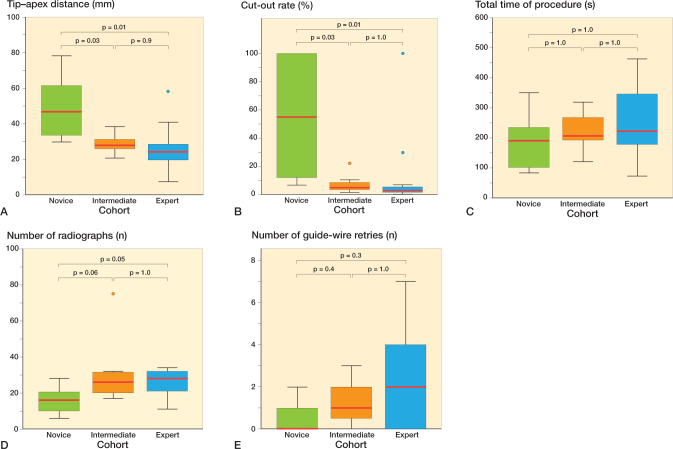
A series of box plots for each objective metric. The central line represents the median, the boundaries of the box represents the upper and lower quartiles respectively, and the whiskers represent the range without outliers. A significance value is presented from the adjusted Mann–Whitney U comparison.

**Objective metrics**—All of the data underwent normality testing. TAD and total procedural time were normally distributed; however, the other 3 metrics were not-normally distributed. For this reason, to allow for standardized comparison between metrics, all statistical analyses used non-parametric methods. The Kruskal–Wallis test compared the distribution between all cohorts at α = 5%. Mann–Whitney U post-hoc testing was used when the Kruskall–Wallis test reached significance. Correction for multiplicity was needed due to the multiple comparisons of the groups. To correct for multiplicity, we multiplied the p-values obtained from each Mann–Whitney U comparison by 3 to maintain a consistent α cut-off value. Therefore, the corrected α cut-off value remained at p = 0.05. This is reflected in both Table 2 and Figure 4.

**Table 2.** Median performance of each cohort

Performance metrics	Novices	Inter-mediates	Experts	p-value^a^
Tip–apex distance (mm)	47	28	24	0.006
Cut-out rate (%)	55	4.7	2.6	0.007
Procedural time (s)	190	206	222	0.6
No. of radiographs (n)	16	26	28	0.03
No. of guide-wire retries (n)	0	1	2	0.2

a Kruskal–Wallis

**Questionnaires**—Percentages of agreement for each statement assessed content and face validity. A score of 5, 6, or 7 relating to mildly, moderately, or strongly agreeing with the statement was seen as the participant agreeing overall.

### Ethics, funding, and potential conflicts of interest

The project outline was submitted to the Project Evaluation Panel at the Royal National Orthopaedic Hospital. Ethical approval was deemed unnecessary due to the non-clinical nature. Informed consent was gained from all participants.

This project received funding from the Professor A. T. Fripp fund. B. H. van Duren is an NIHR funded clinical fellow in Trauma and Orthopedics. R. A. Wescott received funding assistance from the Goldberg Schachmann and Freda Becker Memorial Fund. There were no conflicts of interest.

## Results

### Demographics

The stage of training was recorded using the number of years since medical school graduation, defined as postgraduate year (PGY):

1. Novice group (n = 8) ranged from PGY2 to PGY5 trainees;
2. Intermediate group (n = 7) ranged from PGY4 to PGY9;
3. Expert group (n = 11) ranged from PGY7 and above.

### Objective metrics

A statistically significant difference in TAD, number of radiographs, and COR was observed between all cohorts (Table 2). The experts and the intermediates significantly outperformed the novices for TAD and COR (Table 3 and Figure 4A–B), with experts achieving the lowest scores for these metrics (Table 2). The novices used the least time, had the fewest number of guide-wire retries, and took significantly fewer radiographs compared with the experts (Table 3 and Figure 4C–E).

**Table 3.** Percentage difference and (p-value) between the 3 cohorts for each objective metric ^a^

Performance metrics	Novices vs. inter- mediates	Novices vs. experts	Inter-mediates. vs. expert
Tip–apex distance (mm)	40 (0.03)	48 (0.01)	13 (0.9)
Cut-out rate (%)	92 (0.03)	95 (0.01)	44 (1.0)
Procedural time (s)	8 (1.0)	14 (1.0)	7 (1.0)
No. of radiographs (n)	39 (0.06)	43 (< 0.05)	7 (1.0)
No. of guide-wire retries (n)	100 (0.4)	200 (0.3)	100 (1.0)

a Adjusted p-value presented from Mann–Whitney U post-hoc testing.

### Face and content validity questionnaire

The questionnaire demonstrated the following:

1. 22/26 participants agreed that both the radiographs produced by FluoroSim and the phantom limb model were realistic.
2. 23/26 participants agreed that the content of the simulation would be useful to teach trainees guide-wire insertion into the hip.
3. 25/26 participants agreed that the surgical equipment used in the simulation was realistic.

## Discussion

### Main findings

This study showed a statistically significant difference in both TAD, COR, and number of radiographs taken between all cohorts. Both experts and intermediates outperformed novices in TAD and COR. We expected experts to be faster, use less fluoroscopy, and have fewer retries at guide-wire insertion. However, the opposite trend was observed, with novice participants using significantly fewer radiographs than experts. Face and content validity were also demonstrated.

### Comparison with current literature

Our study showed a statistically significant difference between novices and the other cohorts for TAD and COR (i.e., construct validity), but it was unable to differentiate between intermediate and expert surgeons. This was not unexpected, as it is harder for assessment systems to discriminate between levels of higher skill (Munz et al. 2004). BoneDoc is a computer-based VR DHS simulator that showed similar findings, being able to differentiate medical students from trainee surgeons, but not different levels of trainee surgeons (Blyth et al. 2008).

Another VR DHS simulator, TraumaVision (Swemac Simulation AB, Linkoping, Sweden), demonstrated construct validity. However, intermediates achieved the lowest TAD and COR, possibly due to skills decay of experts and lack of surgical experience of novices (Akhtar et al. 2015). Expert surgeons tend to experience skills decay, from no longer leading the trauma lists and reduced exposure to the DHS procedure to allow residents and fellows (intermediates) to gain more experience.

Using TraumaVision again, a different research group demonstrated that senior surgeons used more guide-wire retries than junior surgeons on the DHS module (Pedersen et al. 2014). Using a VR-based drilling simulator, senior surgeons were shown to take a longer time to complete a task; however, they made fewer mistakes compared with their junior colleagues (Vankipuram et al. 2010). These results were similar in our study. Experts took more time, using more radiographs and more guide-wire retries. However, they still managed to achieve a better TAD and COR compared with novices because experienced surgeons placed more importance on the clinical predictors of DHS failure.

Inexperienced novices placed less importance on obtaining the optimal TAD. Other simulation studies have used an induction period to remove the learning curve of understanding the simulation software (LeBlanc et al. 2013). However, we recorded the first attempt to achieve standardization of our participants using FluoroSim.

### Limitations

This study failed to record the absolute number of DHS procedures completed by each participant individually. Although a cut-off of 40 procedures was selected as this is the number of procedures necessary in the UK to demonstrate competency during formal residency training, we assume 40 procedures as the point of expertise. An additional limitation was the limit of time within the study. Surgeons were asked to participate in between their daily tasks, therefore some participants had a sense of urgency to complete the task, influencing the total procedural time taken. The hand dominance of the surgeon was not accounted for but this procedure required ambidexterity. All participants completed the procedure on a right femur, using their right hand to drill and the left hand to hold the 135-degree angle guide regardless of dominance.

### Future work

Further work is needed to look at the training effect of FluoroSim and the transfer or concurrent validity of FluoroSim in comparison with similar simulators. Ideally, FluoroSim will be used instead of the C-arm in theatre to avoid the risk of radiation exposure. 

## Conclusion

FluoroSim is a useful adjunct in training guide-wire insertion into the hip. It can accurately discriminate between novices and intermediates/experts for clinically validated outcomes in DHS surgery, namely TAD and COR. This is the first of its kind in orthopedic simulation according to current literature. FluoroSim provides an effective and affordable solution to simulate intraoperative imaging without needing radiographs. 

KS proposed the study and the methodology, helped to analyze data as well as helping to write up and review the manuscript, and acting as a project lead co-supervisor. RW collected and analyzed data as well as contributed to writing the manuscript. BvD created the FluoroSim system, reviewed the paper, and acted as project lead co-supervisor. RC contributed to reviewing the paper. AH contributed in reviewing the paper as well as acting as project principal supervisor. 

*Acta* thanks Li Felländer-Tsai and other anonymous reviewers for help with peer review of this study.
